# Evaluation of biofilm formation on acrylic resins used to fabricate dental temporary restorations with the use of 3D printing technology

**DOI:** 10.1186/s12903-022-02488-5

**Published:** 2022-10-13

**Authors:** Justyna Mazurek-Popczyk, Adam Nowicki, Katarzyna Arkusz, Łukasz Pałka, Anna Zimoch-Korzycka, Katarzyna Baldy-Chudzik

**Affiliations:** 1 Department of Microbiology and Molecular Biology, Institute of Health Sciences, Collegium Medicum, University of Zielona Góra, Zielona Góra, Poland; 2 Dental Clinic Diamante, Lubin, Poland; 3 Department of Biomedical Engineering, Faculty of Mechanical Engineering, Institute of Materials and Biomedical Engineering, University of Zielona Góra, Zielona Góra, Poland; 4 Private Dental Practice, Żary, Poland; 5 The Faculty of Biotechnology and Food Science, Wrocław University of Environmental and Life Sciences, Wrocław, Poland

**Keywords:** Provisional restorations, 3D printing technology, Acrylate resins, Biofilm

## Abstract

**Background:**

Temporary implant-retained restorations are required to support function and esthetics of the masticatory system until the final restoration is completed and delivered. Acrylic resins are commonly used in prosthetic dentistry and lately they have been used in three-dimensional (3D) printing technology. Since this technology it is fairly new, the number of studies on their susceptibility to microbial adhesion is low. Restorations placed even for a short period of time may become the reservoir for microorganisms that may affect the peri-implant tissues and trigger inflammation endangering further procedures. The aim of the study was to test the biofilm formation on acrylamide resins used to fabricate temporary restorations in 3D printing technology and to assess if the post-processing impacts microbial adhesion.

**Methods:**

Disk-shaped samples were manufactured using the 3D printing technique from three commercially available UV-curable resins consisting of acrylate and methacrylate oligomers with various time and inhibitors of polymerization (NextDent MFH bleach, NextDent 3D Plus, MazicD Temp). The tested samples were raw, polished and glazed. The ability to create biofilm by oral *streptococci* (*S. mutans*, *S. sanguinis, S. oralis, S. mitis*) was tested, as well as species with higher pathogenic potential: *Staphylococcus aureus, Staphylococcus epidermidis* and *Candida albicans*. The roughness of the materials was measured by an atomic force microscope. Biofilm formation was assessed after 72 h of incubation by crystal violet staining with absorbance measurement, quantification of viable microorganisms, and imaging with a scanning electron microscope (SEM).

**Results:**

Each tested species formed the biofilm on the samples of all three resins. Post-production processing resulted in reduced roughness parameters and biofilm abundance. Polishing and glazing reduced roughness parameters significantly in the NextDent resin group, while glazing alone caused significant surface smoothing in Mazic Temp. A thin layer of microbial biofilm covered glazed resin surfaces with a small number of microorganisms for all tested strains except *S. oralis* and *S. epidermidis,* while raw and polished surfaces were covered with a dense biofilm, rich in microorganisms.

**Conclusions:**

UV-curing acrylic resins used for fabricating temporary restorations in the 3D technology are the interim solution, but are susceptible to adhesion and biofilm formation by oral *streptococci*, *staphylococci* and *Candida*. Post-processing and particularly glazing process significantly reduce bacterial biofilm formation and the risk of failure of final restoration.

**Supplementary Information:**

The online version contains supplementary material available at 10.1186/s12903-022-02488-5.

## Background

Edentulism impacts well-being and life quality [1] and despite taking advanced preventive measures, it still remains a major health problem for dental practitioners. Treatment options vary from complete dentures, implant-retained overdentures to implant- supported fixed restorations [2]. In partial edentulism provisional solutions include single tooth restorations or several-point bridges that may be either removable or fixed [3]. All of these types of restorations may be fabricated with the use of digital workflow [4]. Planning and design of such a treatment protocol is performed individually on a case-by-case basis. This also applies to temporary restorations used for the time of osseointegration of dental implants or before delivering final prosthetic appliance. After successful osseointegration, the provisional restorations are replaced with definitive ones. The shape, colour and short circuit conditions can be individualized, which enables the transfer of precise information regarding the arrangement of the stomatognathic system, the shape of teeth and their macro and microstructure to the technical laboratory responsible for delivering final prosthetic restoration.

Additive technology has enabled manufacturing dental prosthodontic designs with the use of fully digitalized protocols, which include: diagnostics with the use of cone-beam tomography (CBCT), face scan, intraoral scans, as well as data from the registration of movements in the masticatory system. 3D printing is a universal technique with a wide range of materials applied including metal, ceramic and polymers such as resins [5]. Acrylic- and methacrylic-based resins are most commonly used in prosthetics [6]. They have a well-documented history of being used as dental biomaterials due to their good esthetic results, adequate strength, low solubility, and low water sorption. Moreover, they are stable in the oral cavity and have low cytotoxicity [7]. Printable resins consist of photosensitive (also optional thermosetting) liquid monomers. Printed elements are post-cured in an ultraviolet (UV) oven to obtain additional cross-linking of the unreacted monomer chemical groups and improve their mechanical properties [8]. After postprocessing, the material can be used in the raw form but also the manufactured product may be finished using conventional dental methods and instruments: polishing or glazing [9].

The oral cavity is inhabited by a specific microbiota that tends to colonize the surfaces of teeth, tongue and oral mucosa. Microorganisms colonizing the oral cavity live mainly in clusters that form biofilms. In a biofilm, cells are immersed in an extracellular polysaccharide matrix and the structure adheres strongly to the surface. One of the best-known biofilms is dental plaque [10]. Supragingival plaque is mainly formed by Gram-positive bacteria, including *Streptococci* and *Lactobacillus*, while subgingival plaque is dominated by Gram-negative anaerobic bacteria, such as *Fusobacterium*, *Porphyromonas* [10, 11]. The formation of biofilms on the tooth surfaces leads to periodontal diseases and caries. Oral *streptococci* like *Streptococcus sanguinis*, *Streptococcus mutans, Streptococcus oralis* and *Streptococcus mitis* are considered the pioneer colonizers in the formation of dental plaque [12]. They are also capable of colonizing dental implants and have been shown to adhere to dental materials [13–17]. Moreover, biofilms formed on dentures may have even higher contents of *Streptococcus mutans, Streptococcus mitis* and *Streptococcus oralis*, compared to dental plaque [18].

Although the oral cavity is not a typical habitat for *S. aureus*, it is often present in the oral cavity [19]. The current findings indicate that *S. aureus* can constitute a part of a supragingival biofilm [20] and has the ability to incorporate the biofilm formed by *S. mutans* and *S. oralis* [21]. *S. aureus* can be involved in biofilm architecture and trigger the change from a homeostatic biofilm to a dysbiotic biofilm that may lead to the development of oral diseases [22]. What is particularly important is the fact that it has been associated not only to *S. aureus* but also *Staphylococcus epidermidis* and peri-implantitis [19, 23]. *Candida albicans* may also be present in the biofilm of subgingival plaque and oral cavity of individuals with gingival-periodontal disease [24]. An increase in *S. aureus* and *Candida* prevalence was observed in patients who used dental appliances [20, 25]. Adhesion of *Candida* to acrylic is considered a critical factor in the development of denture stomatitis [26, 27].

The microbial biofilm on the abiotic surfaces can be already observed after 24 h and the mature biofilm is obtained within 72 h [28]. Therefore, temporary restorations susceptible to the adhesion of microorganisms can become covered with biofilm and become the source of microorganisms that affect the surrounding tissues and cause implant failure. Moreover, dental materials with a reduced susceptibility to bacterial colonization may have an impact on overall oral health. There is not much information whether temporary restorations manufactured from 3D-printed polyacrylamide resins are susceptible to bacterial adhesion and biofilm formation. This in vitro study aimed to assess the ability to create biofilm by oral *streptococci* (*S. mutans, S. oralis, S. sanguinis*, *S. salivarius*), *staphylococci* and *Candia* on the surfaces of three commercial UV-curable polyacrylamide resins used to fabricate 3D-printed temporary restorations. To assess whether the produced materials require additional processing to reduce any possible biofilm formation, raw, polished and glazed surfaces were tested. Since the surface roughness is an important factor in the adhesion of cells to the surface prior to biofilm formation atomic force microscope was used to test the samples topology. Biofilm biomass was tested by quantitative analysis of crystal violet staining and scanning electron microscopy depicted the resulting biofilms.

## Methods

### Resin characteristics and disks preparation

Three types of materials were used in this study: NextDent MFH bleach, NextDent 3D Plus (NextDent B.V., The Netherlands), and Mazic D Temp (Vericom CO, Corea). These are UV-curable resins used to fabricate 3D-printed crowns and bridges, as well as removable prosthetic restorations. All these resins are CE certified light-curing micro-hybrids. According to the producers, they are biocompatible medical-grade II non-toxic, non-mutagenic, non-sensitizing materials with a good opaque-translucent balance.

NextDent MFH bleach chemical is composed of mequinol; 4-methoxyphenol; hydroquinone monomethyl ether, silicon dioxide, titanium dioxide, 2-hydroxyethyl methacrylate, ethylene dimethacrylate, ethylene dimethacrylate, ethoxylated bisphenol A dimethacrylate, 7,7,9 (or 7,9,9)-trimethyl-4,13-dioxo-3,14-dioxa-5,12-diazahexadecane-1,16-diyl bismethacrylate, 7,7,9 (or 7,9,9)-trimethyl-4,13-dioxo-3,14-dioxa-5,12-diazahexadecane-1,16-diyl bismethacrylate. Monomers constitute acrylate and methacrylate oligomers; a crosslinking agent was phosphine oxide and mequinol served as a polymerization inhibitor. The polymerization time is 30 min. It is characterized by the flexural strength of 107 MPa, sorption 54 μg/mm^3^, solubility 5.9 μg/mm^3^, easy to be polished and dyed to colour.

NextDent 3D Plus consists of silicon dioxide, titanium dioxide, ethoxylated bisphenol A dimethacrylate, diphenyl (2,4,6- trimethylbenzoyl) phosphine oxide, diphenyl (2,4,6- trimethylbenzoyl) phosphine oxide, 2-hydroxyethyl methacrylate, 7,7,9 (or 7,9,9)-trimethyl-4,13-dioxo-3,14-dioxa-5,12-diazahexadecane-1,16-diyl bismethacrylate, 7,7,9 (or 7,9,9)-trimethyl-4,13-dioxo-3,14-dioxa-5,12-diazahexadecane-1,16-diyl bismethacrylate. Monomers constitute acrylate and methacrylate oligomers with a crosslinking agent phosphine oxide. Average polymerization time is 30 min. The flexural strength of this material is 84 MPa, whereas sorption and solubility are 28 μg/mm^3^ and 0.1 μg/mm^3^, respectively.

The third type of material- Mazic D Temp is composed of monomers of methacrylic oligomers, crosslinked by phosphine oxide. The resin is characterized by bending strength of > 50 MPa, sorption 40 μg/mm^3^, and solubility < 7.5 μg/mm^3^.

Printouts were carried out in accordance with the manufacturers' guidelines. The resin bottle was shaken by hand for 5 min and then placed in NextDent LC-3DMixer for an hour. The ambient printing temperature was between 18 and 28 °C. The sample disks were 10 mm in diameter and 4 mm in thickness. The disks from NextDent resins were printed in a NextDent 5100 printer (NextDent B.V., The Netherlands) and samples from Mazic D Temp were printed in Phrozen shuffle 2019 (Phrozen, Taiwan). Post-production took 3 min and consisted of the following steps: washing in an ultrasonic cleaner with organic solvent- isopropyl alcohol, another washing in another batch of > 90% ethanol for 2 min. After 10 min of ethanol evaporation, a 30 min irradiation was performed in the NextDent LC-3DPrint Box UV chamber to obtain the complete conversion of unreacted monomer and gain the additional strength [29]. After the supports were removed mechanically, the first group of tested pulleys was ready for the test.

The next two groups had conditioned polished and glazed surfaces. Polishing of the grain surfaces was performed with the use of a micromotor and a decreasing abrasion gradient of prosthetic rubbers and finally EVE Diacomp paste (EVE Ernst Vetter GmbH, Germany) with the use of a brush with synthetic bristles on the contra-angle. The glazed group, after post-processing, was cleaned with 90% ethanol, and after evaporation, it was veneered with light-polymerizing GC Optiglaze, containing methyl methacrylate monomers (GC N.V. Europe, Belgium). Then, they were irradiated with a LED lamp with a wavelength of less than 430 nm for 40 s. In total, nine types of disk-shaped samples (raw, polished and glazed from three types of resins) were used for the study.

### Microscopic analysis of tested samples

Atomic Force Microscope (AFM) was used to measure the surface roughness of the samples fabricated from the UV-curing resins. All samples were evaluated at the same scan size (5 × 5 μm) by quintuplicating in different areas- all selected randomly- and the mean roughness and peak-to-valley depth (profile) were obtained for each sample. The UV-curing resin surface roughness evaluations were carried out using an AFM (Nanosurf Easy Scan 2, SPM Electronics, Liestal, Switzerland) in the contact mode with a rectangular pyramidal-tipped SICONA-10 contriver (AppNano, USA). The conditions used for the short cantilever contact mode were as follows: spring constant, 0.1–0.6 N/m; resonant frequency 11–19 kHz; length 450 μm; mean width 49 μm; thickness 2.5 μm; tip height 14 μm; radius < 10 nm. The feedback gains with a set point of 20 nN were as follows: P-Gain: 10,000; I-Gain: 1000; and D-Gain: 0. The Nanosurf Easy Scan 2 software (Version 3.10.0) was used to measure the AFM parameters. Surface roughness was quantified by the arithmetic mean (Sa) and the root mean square value (Sq) of the absolute values of the scanned surface roughness profile and the depth profile (peak-to-valley height, Sy) represents the mean of the absolute heights of the five highest profile peaks to the five deepest valleys within the sampling length on the scanned surface.

### Biofilm formation assays

Standard reference strains of *Streptococcus mutans* ATCC 25175, *Streptococcus sanguinis* ATTC 10556, *Streptococcus mitis* NCIMB 13770, *Streptococcus oralis* ATCC 6249, *Staphylococcus aureus* ATCC 29213, *Staphylococcus epidermidis* ATCC 35984 and *Candida albicans* ATCC10231 with a proven biofilm formation capacity were used in this study. All strains were initially cultured on Columbia sheep 5% blood agar (24 h at 37 °C) to obtain single colonies. Next, the inoculum was prepared. *Cocci* strains were suspended in Brain Heart Infusion (BHI) medium (Oxoid, Thermo Scientific) supplemented with 50 mM glucose [30] and *C. albicans* in the Sabouraud Dextrose Medium with 1% glucose (Oxoid, Thermo Scientific) to the optical density OD_600_ = 0.025 ± 0.005 (NanoPhotometerNP60; Implen, Germany). Tested disks were placed in the individual wells of the flat-bottom polystyrene plate (Nest Scientific Biotechnology) and flooded with 1 ml of bacterial inoculum. Cultures were conducted for 72 h at 37 °C with gentle shaking at 50 rpm (ES20 Biosan, Latvia), and the medium was changed (refreshed) every 24 h. After incubation, disks were carefully washed twice with 1 mL of phosphate-buffered saline (PBS; Chempur, Poland) to remove the non-adhered cells and investigated in subsequent tests.

### Quantification of biofilm biomass

The biofilm on the sample surface was stained with crystal violet and then the absorbance of the dissolved dye was tested. The absorbance measurement is proportional to the biofilm abundance present on the sample surface [31]. First, biofilm on the disks was fixed in 1 mL of 10% formalin for 5 min and then rinsed with 1 mL of PBS. Subsequent biofilm was stained by 1 mL of 0.1% crystal violet (POCH, Poland) for 15 min and then an excess of the dye was rinsed three times with PBS. Samples were dried at room temperature for 15 min. The crystal violet from biofilm biomass was solubilized in 1 mL of methanol on a shaking table for one hour. The solution was collected and as needed diluted tenfold with methanol. The absorbance was measured at 590 nm using a spectrophotometer (BioMate, Thermo Scientific, UK) [31]. Sterile disks were used as a negative control. The tests were performed in triplicate.

The quantity of viable microorganisms in biofilm was determined also by CFU (colony forming units) counting. After biofilm formation, the disks were carefully washed three times with 1 mL of PBS to remove the unattached cells. Next, they were put in tubes containing 1 mL of PBS, vigorously vortexed for 2 min and sonicated twice for 10 s to disperse cells attached on the surface of disks [30]. Cell suspension from each sample was serially ten-fold diluted in PBS and plated onto Columbia sheep 5% blood agar. The cultures were incubated at 37 °C for 24 h, s*treptococci* cultures additionally, were grown in the atmosphere of 5% CO_2_ using GasPak (Oxoid, Thermo Scientific UK). The grown colonies were counted and the total number of viable cells was expressed as CFU/mL. All experiments were performed in triplicate.

### Microscopic imaging of biofilm

The preparation of samples contained fixation with glutaraldehyde, dehydration, drying, and mounting [32]. First, the disks covered with biofilms were immersed in 3% glutaraldehyde (25% in H_2_O, stage I) in phosphate-buffered saline (15 min), then in acetone solutions (concentration increases gradually from 10 to 100% increment 10% for 10 min at each concentration). Finally, the samples were dried using a CPD E3000 critical point dryer (Quorum Technologies, UK) and sputter-coated with a chromium layer of about 10 nm. The specimens were examined in a JEOL JSM-6490LV Scanning Electron Microscope (JEOL, Japan).

### Statistical data analysis

Statistical data analysis was performed using Statistica 12 (StatSoft, Krakow, Poland) by examining the influence of two factors (type of material and type of post-production method) on biofilm biomass formation. Variance analysis was used and the differences between the meanings were determined by the Duncan test with a significance level of *p* < 0.05.

## Results

### Characteristics of the surface topology

**Fig. 1:**
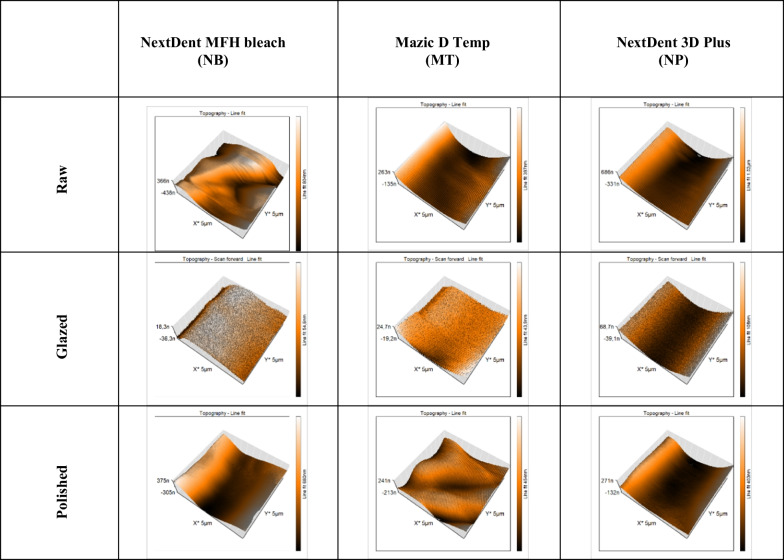
Representative 3-D images show the surface roughness of disks from UV-curing resin differing in material and surface treatment

The roughness measurements of three types of the photopolymer methacrylate resins NextDent MFH bleach (NB), Mazic D Temp (MT), NextDent 3D Plus (NP) varying by surface treatment, obtained with contact mode AFM allowed to obtain the representative 3-D AFM map and surface roughness parameters, presented in Fig. 1 and Table 1, respectively. The highest value of the roughness average (Sa) was obtained for each type of raw sample not subjected to surface modification. Among them, the highest Sa was measured in samples of raw acrylate/methacrylate resins NB (273.78 ± 80.79 nm) and NP (209.77 ± 40.59 nm), the lowest Sa value was 70.75 ± 5.53 nm recorded for Mazic D Temp - methacrylic resin. Polishing caused Sa reduction for each analyzed sample, but the most significant reduction was observed for the glazed specimens. By comparing surface materials, the Sa range was between 5.32 ± 2.11 nm and 21.46 ± 9.46 nm for three tested types of glazed photopolymer resin, and between 66.59 ± 5.77 nm and 134.54 ± 15.92 nm for the polished samples. Other roughness parameters, Sq (the root mean square) and Sy (the peak valley high) values, showed a greater dispersion of results for all three raw materials (Table 1).

**Table 1:** Roughness profile values of disks from UV-curing resins differing in material and surface treatment, measured across a 5 × 5 µm area, with SD – standard deviation

Resin type	Post-processing method	Sa* ± SD [nm]	Sq* ± SD [nm]	Sy* ± SD [nm]
NextDent MFH bleach (NB)				
	Raw	273.78 ± 80.79	344.14 ± 103.97	1865.72 ± 509.91
	Polished	134.54 ± 15.92	166.89 ± 11.49	970.15 ± 160.32
	Glazed	5.35 ± 2.11	6.85 ± 2.61	157.12 ± 189.97
Mazic D temp (MT)				
	Raw	70.75 ± 5.53	87.61 ± 50.76	485.16 ± 41.05
	Polished	67.8978 ± 7.45	80.6422 ± 8.29	543.264 ± 44.69
	Glazed	21.46 ± 9.46	28.01 ± 13.32	339.44 ± 167.80
NextDent 3D plus (NP)				
	Raw	209.77 ± 40.59	262.95 ± 57.45	1644.58 ± 472.28
	Polished	66.59 ± 5.77	81.16 ± 5.64	482.40 ± 92.41
	Glazed	15.92 ± 3.82	18.73 ± 4.21	120.47 ± 18.67

^*^Sa, roughness average; *Sq*, the root mean square value of ordinate values within the defined area; *Sy*, peak-to-valley height -the difference between the highest and lowest peak value within the defined area

### Quantitative analysis of biofilm formation

Quantitative analysis of biofilm biomass showed that after 72 h all tested strains formed the biofilm on the surface of the tested resin disks. Biofilm biomass on the raw materials dominated. The highest values in the measurements of biomass staining were noted for *S. oralis* and *S. sanquinis* biofilm as well as for *Candida albicans* (Table 2). The largest number of living cells grown from biofilm biomass was observed for *S. oralis*, *S. aureus* and *S. epidermidis* (Table 3). It was noted that the glazing process caused significant reduction of bacterial biofilm on all types of the tested disks (Tables 2, 3). The lowest biofilm biomass of *S. mitis* (0.5), *S. mutans* (0.43), *S. sanquinis* (1.43) and *S. aureus* (1.57) were noted for a glazed disk and NextDent MFH bleach (NB) (Table 2). Biofilm biomass of *S. oralis* (8.33) and *C. albicans* (1.07) were the lowest for Mazic D Temp glazed disks (MT). The exception was noted for the growth of *S. epidermidis*, where the lowest absorbance values were observed for biofilm on glazed NextDent 3D Plus disks (NP).

**Table 2:** Quantitative analysis of biofilm biomass based on absorbance values with statistical analysis of the material type and post-processing method on biofilm biomass

Resin type	Post-processing method	*S. mutans*	*S. sanquinis*	*S. mitis*	*S. oralis*	*S. aureus*	*S. epidermidis*	*C. albicans*
NextDent MFH bleach (NB)								
	Raw	2.25^b^	12.46^b^	6.73^c^	22.33^c^	15.90^c^	8.82^c^	21.25^bc^
	Polished	1.29^ab^	7.06^ab^	2.65^ab^	17.77^ac^	5.60^b^	4.47^b^	6.99^d^
	Glazed	0.43^a^	1.43^a^	0.50^a^	9.68^ab^	1.57^a^	3.67^ab^	1.90^a^
Mazic D temp (MT)								
	Raw	2.19^b^	12.76^b^	2.67^ab^	18.27^ac^	10.22^c^	2.08^ab^	19.91^b^
	Polished	2.26^b^	6.39^ab^	1.01^a^	14.97^abc^	7.57^bc^	1.68^ab^	13.24^f^
	Glazed	0.46^a^	3.06^a^	0.65^a^	8.33^b^	1.76^a^	1.47^ab^	1.07^a^
NextDent 3D Plus (NP)								
	Raw	6.66^c^	3.15^a^	8.25^c^	17.60^ac^	13.37^c^	1.80^ab^	22.30^c^
	Polished	2.53^b^	2.37^a^	5.62^bc^	13.67^ab^	8.20^bc^	1.54^ab^	10.84^e^
	Glazed	0.66^a^	2.07^a^	0.97^a^	10.16^ab^	2.30^a^	0.54^a^	2.62^a^

Values with different letters (a–f) within the same column differ significantly (*p* < 0.05)

**Table 3:** The quantity of viable microorganisms in biofilms expressed by colony forming units (CFU/mL) with statistical analysis of the material type and post-processing method on biofilm biomass

Material type	Post-processing method	*S. mutans*	*S. sanquinis*	*S. mitis*	*S. oralis*	*S. aureus*	*S. epidermidis*	*C. albicans*
NextDent MFH bleach (NB)								
	Raw	6.83E+05^ cd^	4.05E+06^bc^	5.29E+06^bc^	2.40E+07^e^	4.73E+07^b^	2.42E+08^b^	2.28E+04^a^
	Polished	4.65E+05^bc^	2.78E+06^abc^	4.33E+05^a^	1.87E+07^de^	1.76E+07^a^	1.89E+08^ab^	2.62E+08^c^
	Glazed	1.84E+05^a^	1.16E+06^a^	3.04E+04^a^	9.20E+06^ab^	1.43E+07^a^	1.65E+08^ab^	3.14E+08^c^
Mazic D temp (MT)								
	Raw	2.40E+05^ab^	4.20E+06^c^	6.91E+05^a^	1.79E+07^cde^	3.45E+07^c^	1.48E+08^ab^	3.54E+08^c^
	Polished	6.22E+05^ cd^	3.24E+06^abc^	3.73E+04^a^	1.65E+07^bcde^	2.24E+07^a^	1.31E+08^ab^	9.07E+07^b^
	Glazed	1.47E+05a	1.60E+06^ab^	2.10E+04^a^	7.97E+06^a^	1.92E+07^a^	9.73E+07^a^	5.99E+07^b^
NextDent 3D plus (NP)								
	Raw	1.44E+06^e^	2.15E+06^abc^	6.48E+06^c^	1.53E+07^abcd^	4.49E+07^b^	1.43E+08^ab^	5.14E+08^c^
	Polished	7.10E+05^d^	1.55E+06^ab^	3.73E+06^b^	1.35E+07^abcd^	4.13E+07^bc^	1.22E+08^a^	5.87E+07^b^
	Glazed	2.96E+05^ab^	1.39E+06^a^	2.56E+04^a^	1.06E+07^abc^	1.36E+07^a^	8.80E+07^a^	1.63E+05^a^

Values with different letters (a–e) within the same column differ significantly (*p* < 0.05)

The results of counting live cells separated from the biofilm biomass and expressed as CFU/mL were consistent with the measurement of the biomass value regarding the reduction of biofilm on polished and significantly- glazed disks. Some differences were observed in the type of material covered for some species, indicating that the type of material is not a key factor in the effectiveness of microbial growth limitation. The lowest growth of *S. mitis* (2.10E + 04 CFU/mL), *S. aureus* (1.36E + 07 CFU/mL) and *S. epidermidis* (8.80E + 07 CFU/mL) were noted for Nexdent 3D plus (NP) material and glazing method (Table 3). Growth of *S. mutans* (1.47E + 05 CFU/mL) and *S. oralis* (7.97E + 06 CFU/mL) were the lowest for Mazic D Temp material (MT) and glazing method. The lowest number of *S. sanquinis* (1.16E + 06 CFU/mL) was seen for Nexdent MFH bleach material and glazing method (Table 3). The values of CFU/mL of *C. albicans* deviated from the previously observed trend. The lowest values for *C. albicans* were noted for not processed Nexdent MFH bleach disk (NB). At the same time, there were no significant differences between variants NB raw and Nexdent 3D plus (NP) glazed. It seems that growth and inhibition of *C. albicans* is more complicated and more tests need to be performed.

### Visualization of biofilm by scanning electron microscopy

**Fig. 2:**
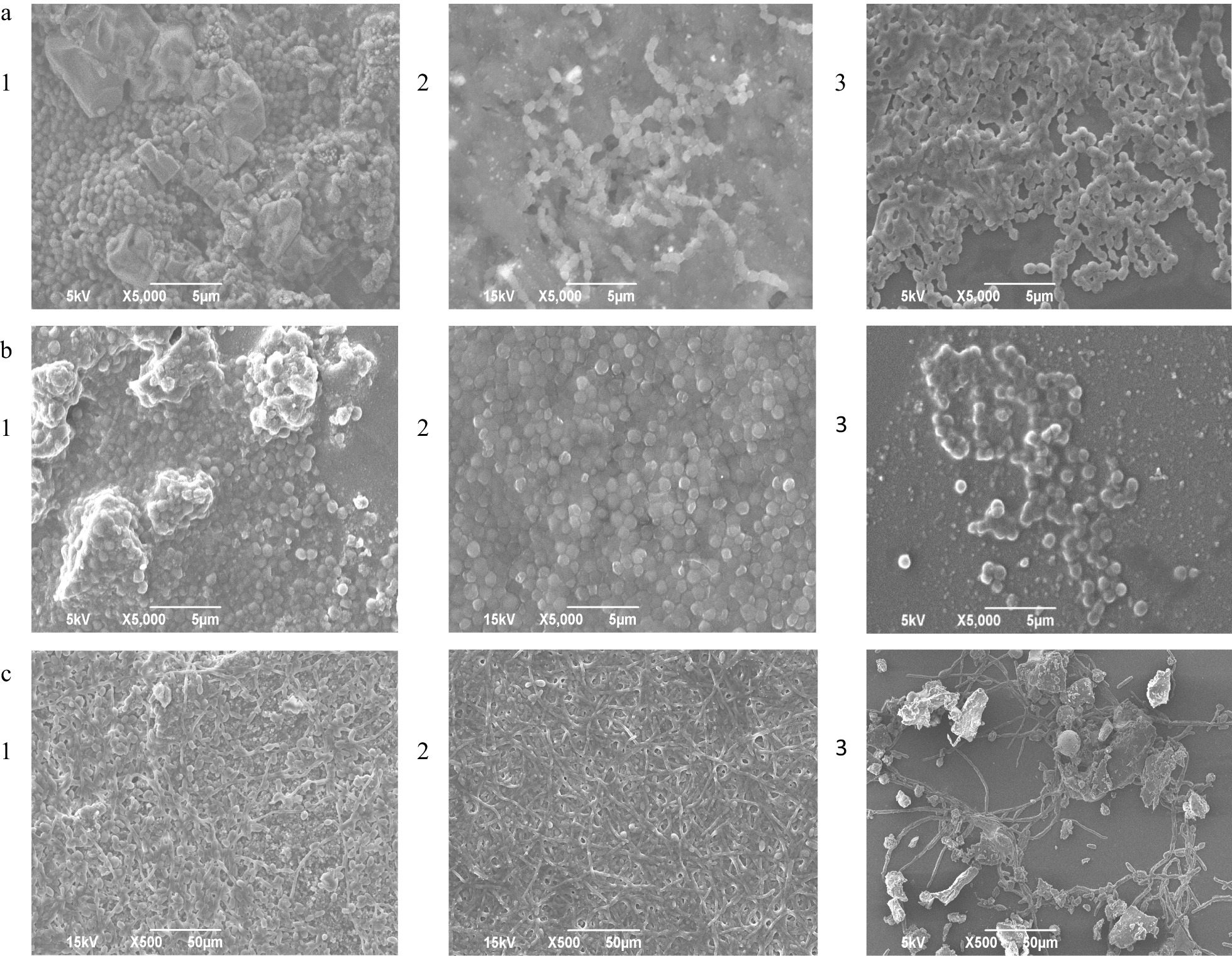
Scanning electron microscopic photographs showing biofilms on the resins disks; **a**: *S. mutans* on Nexdent MFH bleach*;* **b**: *S. aureus* on Nexdent 3D plus; **c**: *Candia albicans* on MazicD Temp; 1 – raw; 2 – polished; 3 – glazed

Scanning electron microscopy allowed to investigate the biofilm structure and confirmed data of quantitative analysis of biofilm formation. Representative SEM photographs are presented for *S. mutans*, *S. aureus* and *Candida albicans* (Fig. 2). The micrographs of biofilm for all the tested strains are included in Additional file 1. The densest biofilm with a visible extracellular matrix formed on the surface of raw resin disks of all three tested materials. This applied to all species tested. The biofilm was also characterized by a more complex, three-dimensional, multilayered architecture compared to the polished disks (Fig. 2). In *C. albicans* biofilm, filaments (hyphae) prevailed over yeast cells and covered tightly the surfaces of the tested resins disks. Slightly less biofilm was observed on the polished disks. The single-layer biofilm on the glazed disks was predominant for *S. mutans*, *S. mitis* and *S. sanquinis* on MT and NP. There, the biofilm was fragmented during fixation, and raw material was exposed in a number of cases. A small amount of exopolysaccharide matrix was observed and bare cells showed the typical arrangement of bacterial cells e.g., chains of *S. mutans*, grape-like clusters of *Staphylococci* and long hyphae of *Candida albicans*. Only *S. oralis* and *S. epidermidis* continued to cover the glazed disks (on all three types of disks and on NB and MT, respectively) with a dense biofilm.

## Discussion

Acrylic resins are the most commonly used polymeric materials in dentistry [33], that recently may be also used to fabricate 3D-printed prosthetic restorations. 3D-printed resins for provisional dental restorations are of interest in numerous ongoing studies [34–37]. However, there are no extensive studies on the biofilm formation on these materials. Microorganisms adhering to dental implants and other prosthetic restorations may be the cause of infection, secondary caries and contribute to pulp pathology [37]. They can also contribute to the material degradation and roughness changes, as was demonstrated for *S. mutans* [10, 38–41]. A pronounced biofilm on biomaterials in the oral cavity is already detectable after 24 h, multispecies biofilm occurs within 3–5 days and biofilm maturation is achieved within 2–3 weeks [13, 42]. Therefore, even in the case of temporary restorations microorganisms on their surface may contribute to the deterioration of the condition of the oral cavity and threaten final reconstruction.

All studied oral *streptococci* as well as *staphylococci* and *Candida* demonstrated the ability to form biofilm on the tested materials. The most abundant biofilm was created on raw materials, less abundant on the polished resins, and glazed surfaces were the least conducive to biofilm formation. These results were consistent with the change in the roughness parameters. Surface roughness is one of the key factors influencing the adhesion and colonization of microorganisms on biomaterials. The depressions in roughened surfaces provide more favorable colonization sites [43, 44]. Post-production treatment may be aimed at smoothing the surface. AFM results obtained in this study indicate that the type of material and surface treatment play a significant role in the roughness of UV-curing resins. The highest roughness average value was measured for raw materials, especially for NB and NP samples, made of identical materials (acrylate and methacrylate oligomers) but differing in the addition of a polymerization inhibitor: mequinol added to NextDent MFH bleach (NB). Thus, the results confirm that the roughness of UV-curving resin depends on the polymerization time, where the extended polymerization time increases the surface roughness [45]. This assumption confirms the lowest Sa value recorded for Mazic D Temp - methacrylic resin with very short polymerization time, i.e., 6 min compared to 30 min for the NextDent group. The polishing technique reduced the roughness average (Sa) for each analyzed sample, but the most significant reduction was observed for glazed samples. Although roughness average is the most commonly used parameter to quantify roughness, it is recommended to use different roughness parameters including amplitude or spacing parameters influencing the bacterial adhesion, optical feature, or other properties [46]. Before surface treatment, Sq, which is sensitive to peaks and valleys on the surface, and Sy, which is more sensitive to the distribution of peaks and valleys, showed a greater dispersion of results for three different materials. After post-processing, lower values of Sq and Sy were measured for each analyzed sample and confirmed the greater homogeneity of the samples over the entire surface and lower affinity for bacterial adhesion and colonization, which confirms previous observations [47, 48]. The procedure of glazing dental restorations is applied to provide better esthetics and results in smoothening rough surfaces by filling the micro-cracks and porosities [49, 50]. This also lowers bacteria's ability to adhere and form biofilm [51]. Our research revealed a significant reduction in biofilm on glazed resins. Glazed surfaces were covered by a thin layer of microbial biofilm and with a small number of microorganisms for all tested strains except *S. oralis.* Sesma et al. revealed in their in vivo study, that even if glazing dentures surface did not prevent bacterial colonization it facilitated the removal of plaque [51]. This is especially important for reducing *Candida* colonization since yeasts adhere quite strongly to acrylic denture base materials and mechanical or chemical removal of fungal biofilms constitutes a significant clinical problem [42, 52].

The effect of roughness on microbial adhesion has been demonstrated for various dental biomaterials including composite resins but manufactured mainly in a traditional way. Mei et al. demonstrated that *Streptococcal* adhesion to orthodontic light-cured composite resins increases with increasing roughness of the composite surfaces. In their studies, composite surface roughness affected more adhesion forces for *S. sanguinis* than *S. mutans* [16]. Also, our research on advanced 3D technology showed greater differences for *S. sanquinis* than for *S. mutans*. The manufacturing technology may therefore not be a discriminating parameter regarding the adhesion of microorganisms. There are only a few studies on bacterial adhesion on 3D-printed polished resins. They show that acrylic resins from different manufacturers have slightly different roughness parameters. Schubert et al. assessed four products used for 3D printing of oral splint acrylic resins and their effect on *Streptococcus mutans* and *Candida albicans* adhesion. Mean roughness values of polished samples ranged from 0.064 to 0.091 µm. Material samples were incubated with these microorganisms for two hours and this time was sufficient for adhesion. A positive, but statistically insignificant, correlation between the surface roughness and adhesion of each microorganism was observed [17]. Our research has shown greater differences, but only mean roughness arithmetical values (Ra) were calculated for the tested resin (by widefield confocal microscopy) in the mentioned studies. Samples printed from acrylic resin were also tested by Al-Fouzan et al. *Candida albicans* adhesion was observed on the polished disks characterized by mean Sa value of 0.037 μm [53]. A slightly different acrylic resin composition and post-production treatment lead to a different surface structure, but the minimum parameters for the adhesion of microorganisms have not been determined.

As the main primary colonizers *streptococci* can create an environment for the adhesion of others microorganisms and allow the formation of complex pathogenic biofilms [22]. *S. oralis* formed the most abundant biofilm on all tested resins and this species is one of the most common species detected on the tooth surface [54, 55]. It can create a biofilm on conventionally processed denture acrylic of different surface roughness and also on brushed dentures with dentifrices [56]. It has been shown that *S. sanguinis* and *S. mitis* are capable of biofilm formation on dental implants and restorative materials manufactured with the use of conventional procedure [15]. What is interesting they can contribute to the settling of other microorganisms, including *C. albicans* adhesion and propagation. *C. albicans* colonization on acrylic dentures is well known and it has been shown that the presence of this yeast significantly affects the health of the oral cavity in people having dentures [25]. Taking into account the results of our research, it can be concluded that the manufacturing method using 3D printing technology itself does not limit the adhesion capacity of this yeast to acrylic resins. It only changes the surface roughness in the post-manufacturing process. Raw and polished samples in our research were covered with a dense biofilm comprised mostly of yeast filaments - more infectious form of *Candida*, since the formation of hyphae and phenotypic switching is involved in the virulence of the fungus [57]. This is an argument in favor of such a post-processing method of printed temporary restorations to limit the formation of microbial biofilm.

Another *streptococcus* used in our research, i.e., *Streptococcus mutans*, formed less abundant biofilm compared to other species tested. *S. mutans* constitutes an important etiologic agent in dental caries. More importantly, *S. mutans* metabolizes dietary sugars (fructose, glucose and sucrose) into lactic acid, which demineralizes tooth surfaces, causing carious lesions [58]. The products of metabolism and low pH may affect the stability of the material to which it adheres. In the case of acrylamide resins, their safety is particularly tested [59, 60]. Surface deterioration of resin composites (bisphenol A glycidyl methacrylate, urethane dimethacrylate) has been demonstrated after a one-month exposure to a *S.mutans* biofilm and increased surface roughness [14, 40]. The research concerned conventional manufacturing technology and revealed an increase in surface roughness from less than 10 to above 40 nm (without affecting the microhardness) suggesting the removal of filler particles based on the roughness dimensions created [40]. In turn, other research conducted by Kim et al. revealed that bisphenol A glycidyl methacrylate, which is released into the oral environment by acrylic resins through incomplete polymerization, hydrolysis or mechanical degradation, could enhance *S. mutans* adhesion to hard surfaces by glucan synthesis and the virulent properties of *S. mutans* [61]. The reports mentioned above emphasize the necessity of taking actions aimed at the production of dental restorations with low susceptibility to *S. mutans* adhesion. The influence of *S. mutans* on the tested resins has not been the subject of our research but microscopic images from SEM analysis showing the brightness around the single of *S. mutans* chains adjacent to the glazed surface (not observed for other tested species) may be an indication for further research.

Biofilm formation of *Staphylococcus aureus* and *Staphylococcus epidermidis* was also the subject of this research. Individuals with periodontal disease represent reservoirs of *S. aureus* and *S. epidermidis* in the oral cavity [24]. *S. aureus* can contribute to the development of several oral diseases including angular cheilitis, staphylococcal mucositis and more importantly, play a role in dental implant failure [62, 63]. Similarly, *S. epidermidis* causes implant-related infections. In the present research *S. aureus* and *S. epidermidis* formed abundant biofilms with a high number of recovered viable cells but reduction of biofilm biomass along with post-processing procedures and decrease in the surface roughness were noticeable. Interestingly, *S. epidermidis* showed a strong affinity for NextDent MFH bleach (NB) resin and covered profusely also polished and glazed disks printed from this resin. To clarify this observation, further analysis of the physical characteristics of the materials should be carried out like the surface free energy related to the wettability and hydrophobicity of the tested surfaces.

The demonstrated ability to create biofilm for each of the tested species is important for health reasons. Temporary restorations from acrylic resins implanted for several days or weeks covered with biofilm can affect the surrounding tissues, cause inflammatory processes and contribute to the eventual failure of implant-based treatments. Tested species also pose the risk of developing systemic infectious diseases. During the preparation of the implantation site, the continuity of the tissues is broken, which may lead to the translocation of pathogenic and/or opportunistic microorganisms towards the bloodstream. This also applies to *streptococci* like *S. sanguinis*, which causes infective endocarditis [64], *S. mitis* as a leading cause of infective endocarditis and bacteremia especially in neutropenic and immune-compromised patients [65]. Also *S. mutans* is linked to the severe medical conditions such as bacterial endocarditis and atherosclerosis [58].

Our in vitro research is based on mono-species biofilms, but strong adhesion of tested microorganisms demonstrated on raw and polished printed samples from acrylic resins constitute an indication for the development of biofilm in vitro. In vivo adhesion patterns of microorganism may differ from this in vitro and this is one of the limitations of the study. The salivary pellicle is an important factor influencing bacterial adhesion in oral cavity [66]. Saliva coating results in a more hydrophilic surface [67] and can increase adherence of microorganisms like *streptococci* [68, 69]. It should be added that although the presented studies used cylindrical disks for standardization of research, they cannot fully reflect the target temporary restorations. Printed temporary restorations are personalized and in the case of dental crown restorations, they have different shapes. Since irregularities and cavities promote the adhesion of microorganisms, in vitro the biofilm formation will be reinforced by macro irregularities formed by cusps and fissures of crowns of the temporary restorations.

## Conclusions

The use of light-cured resins in 3D printing technology enables not only predictable implant treatment from the diagnostic stage to the final restoration, but also their individualization and modification during treatment. Temporary restorations protect the implant site and soft tissues against harmful environment and invasion of external substances and microorganisms and, at the same time, help in recovering tooth functions. Our research has shown that in a short time they can be covered with biofilm formed by microorganisms with different pathogenicity potential. The research indicates that post-production processing such as glazing is very important, as it reduces the surface roughness and consequently the formation of biofilm. For optimal benefits, temporary restorations should be fabricated with great care and attention to serve as a functional and esthetic plan for final restorations but also minimizing bacterial adhesion and their adverse health effects.

## Supplementary Information

**Additional file 1**.** Supplementary Figure 1**. Scanning electron microscopic photographs of microbial biofilms on resins disks.

## Data Availability

The datasets supporting the conclusions of this article are included within the article. Raw datasets are available from the corresponding author on reasonable request.

## Abbreviations

NB: NextDent MFH bleach resin
MT: Mazic temp resin
NP: NextDent 3D plus resin
AFM: Atomic force microscope
SEM: Scanning electron microscope
