# Supplementary material for: Evaluation of biofilm formation on acrylic resins used to fabricate dental temporary restorations with the use of 3D printing technology
Source: BMC Oral Health. 2022 Oct 13;22:442. doi: 10.1186/s12903-022-02488-5 (PMC9563793; doi:10.1186/s12903-022-02488-5)

Supplementary Figure 1. Scanning electron microscopic photographs of microbial biofilms on resins disks

S. mutans
biofilm on disks from:

NB-
Nexdent MFH bleach

MT-
Mazic D Temp

NP-
Nexdent 3D plus

r - raw;
p - polished;
g - glazed

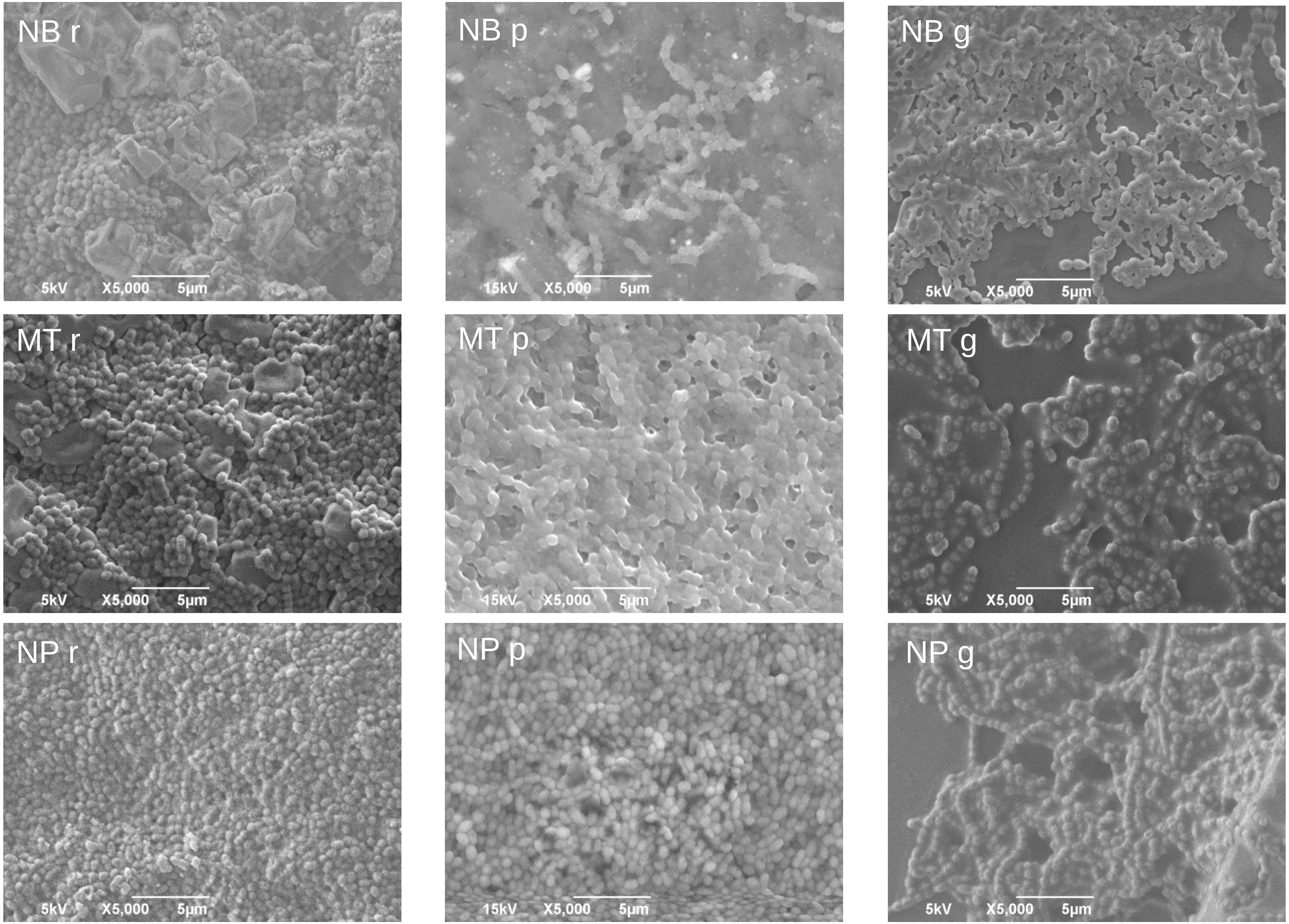

S. sanquinis
biofilm on disks from:

NB-
Nexdent MFH bleach

MT-
Mazic D Temp

NP-
Nexdent 3D plus

r - raw;
p - polished;
g - glazed

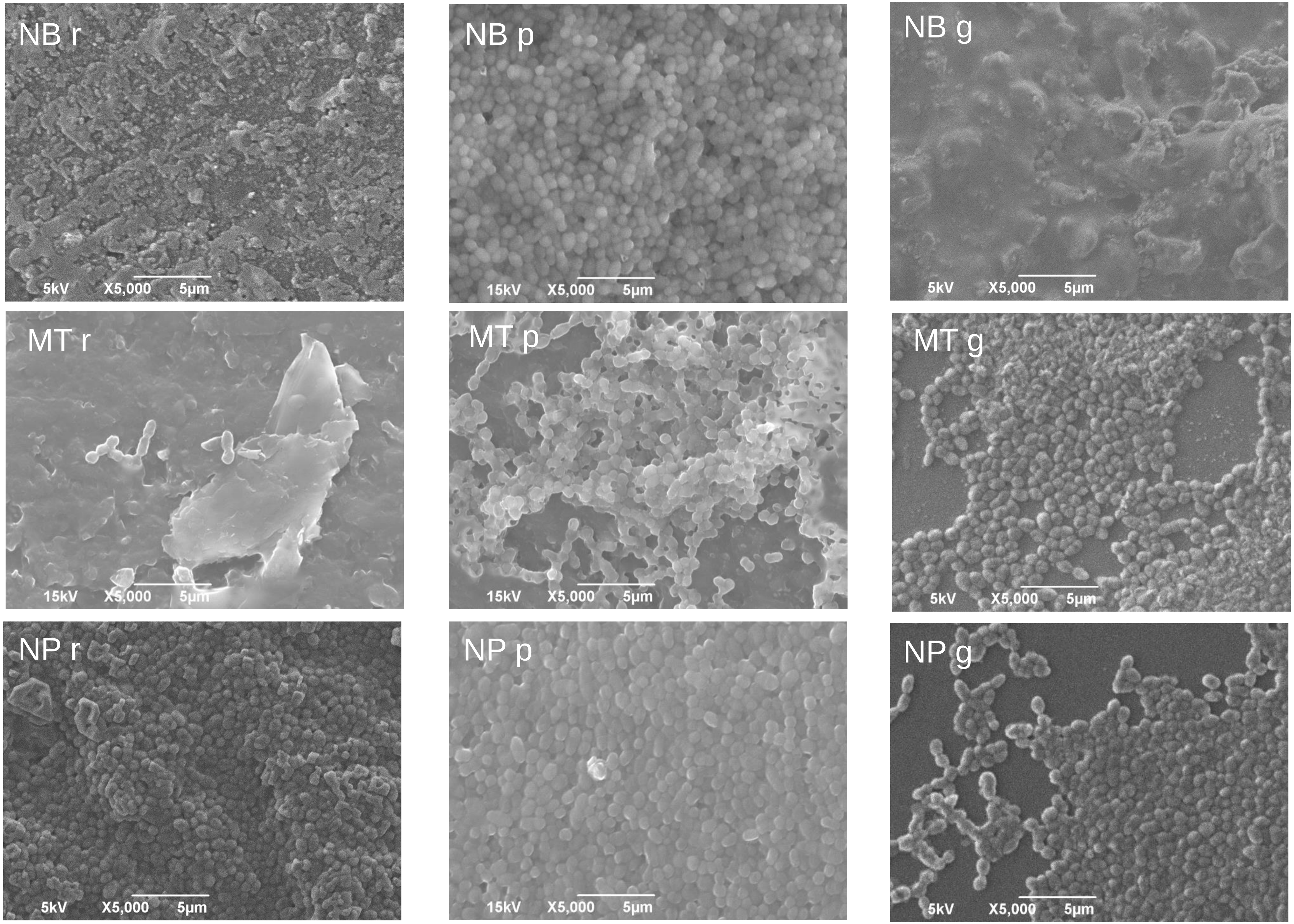

S. mitis
biofilm on disks from:

NB-
Nexdent MFH bleach

MT-
Mazic D Temp

NP-
Nexdent 3D plus

r - raw;
p - polished;
g - glazed

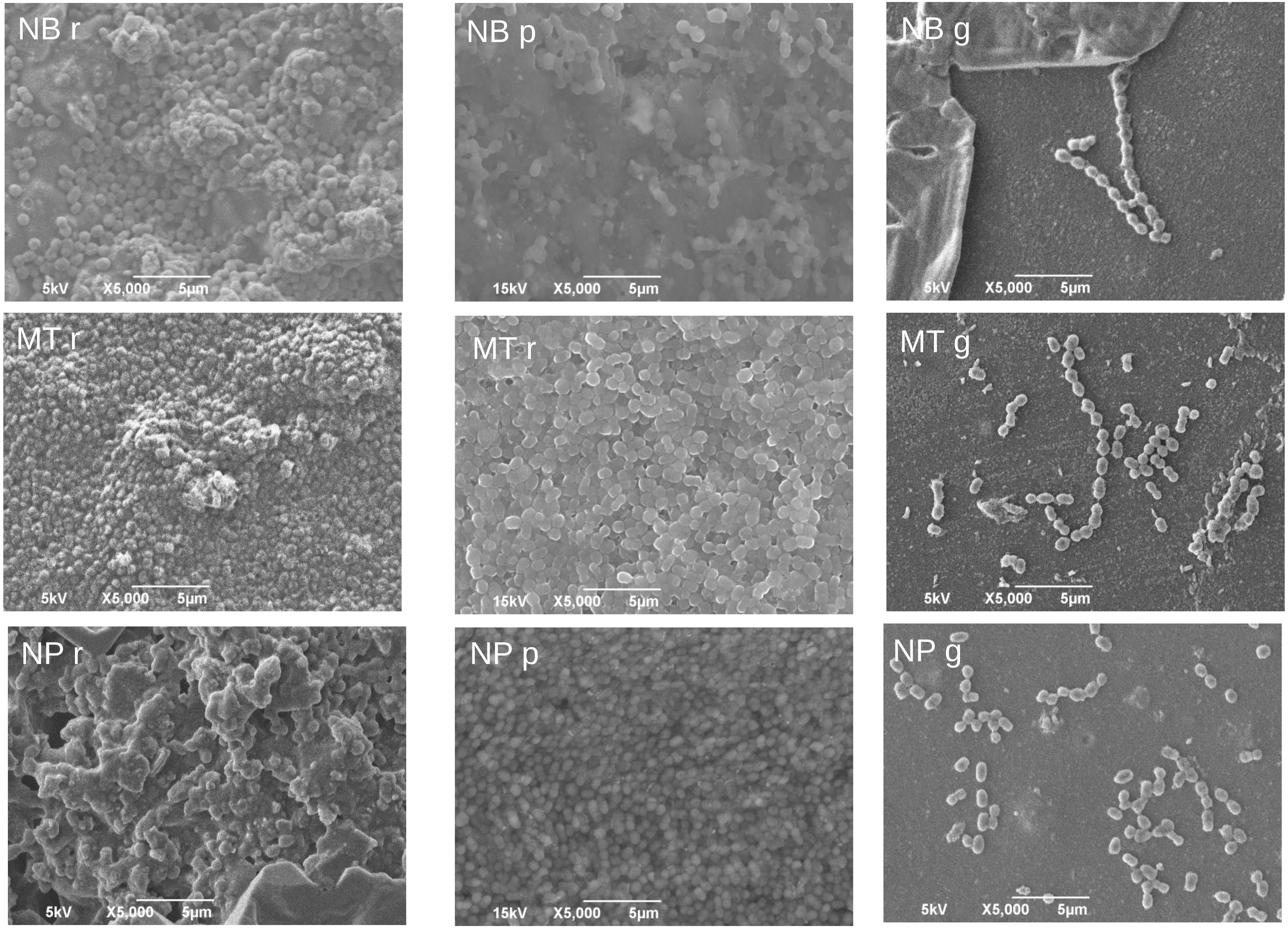

S. oralis
biofilm on disks
from:

NB-
Nexdent MFH bleach

MT-
Mazic D Temp

NP-
Nexdent 3D plus

r - raw;
p - polished;
g - glazed

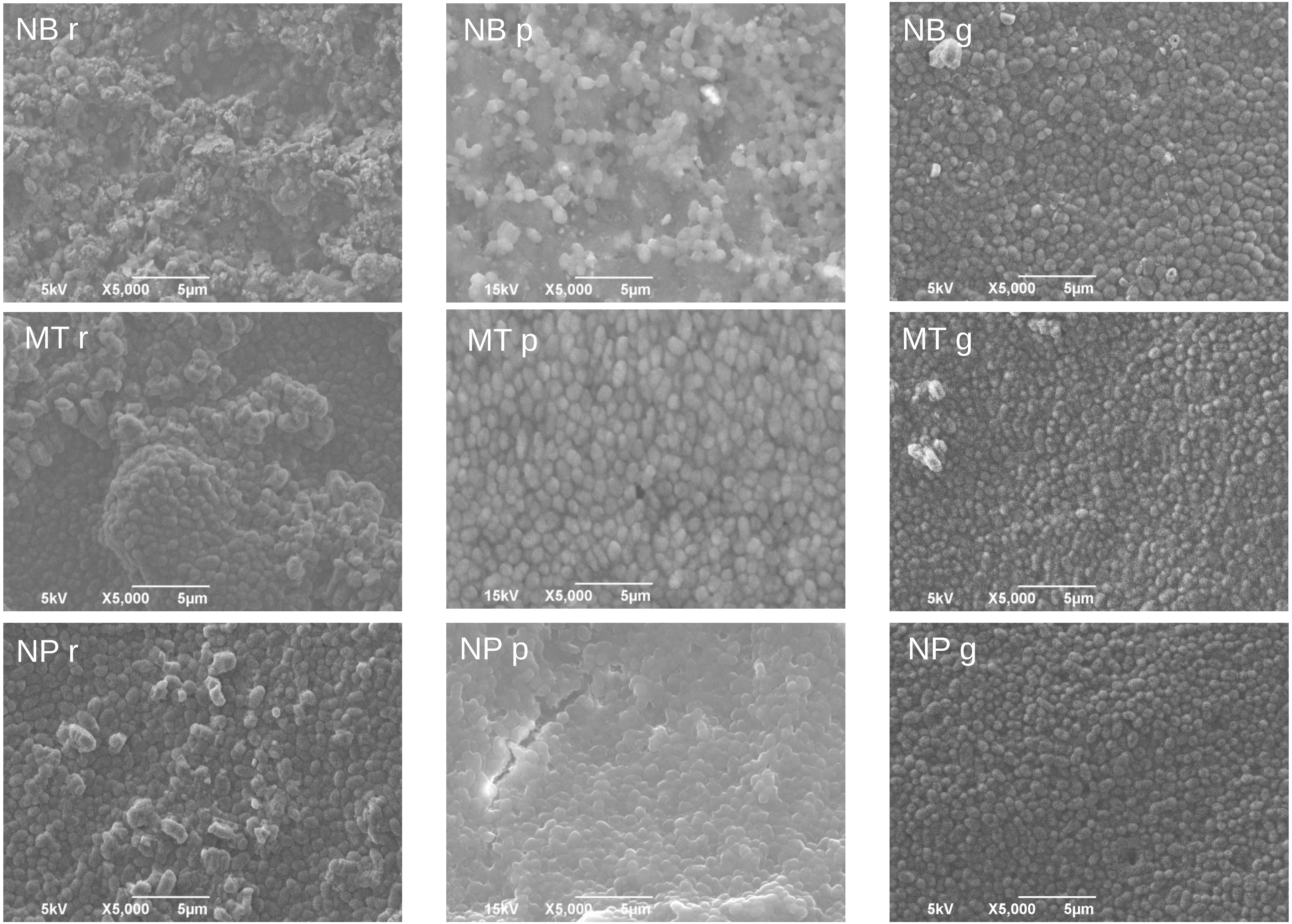

S. aureus
biofilm on disks
from:

NB-
Nexdent MFH
bleach

MT-
Mazic D Temp

NP-
Nexdent 3D plus

r - raw;
p - polished;
g - glazed

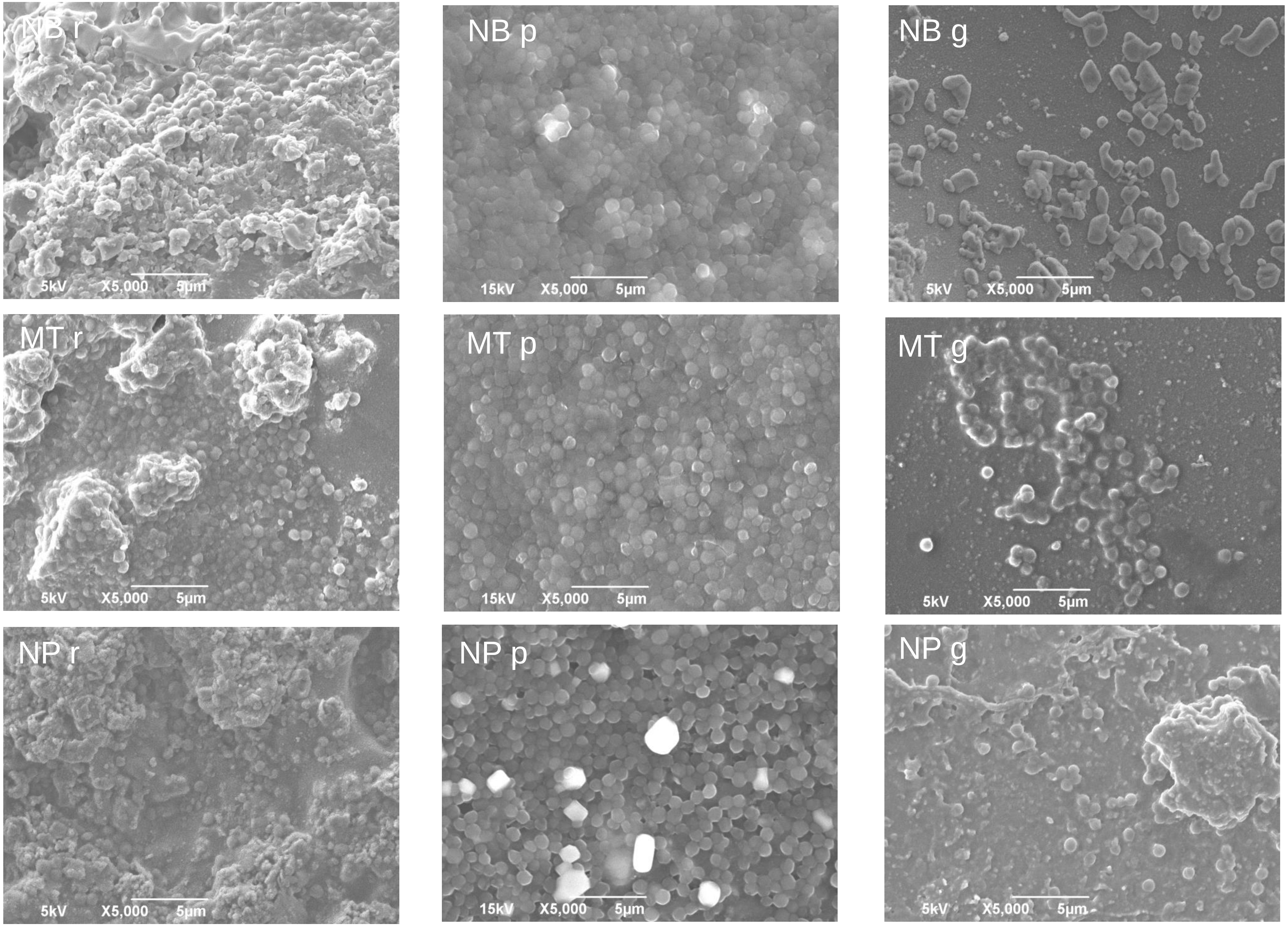

S. epidermidis biofilm on disks from:

NB-
Nexdent MFH bleach

MT-
Mazic D Temp

NP-
Nexdent 3D plus

r - raw;
p - polished;
g - glazed

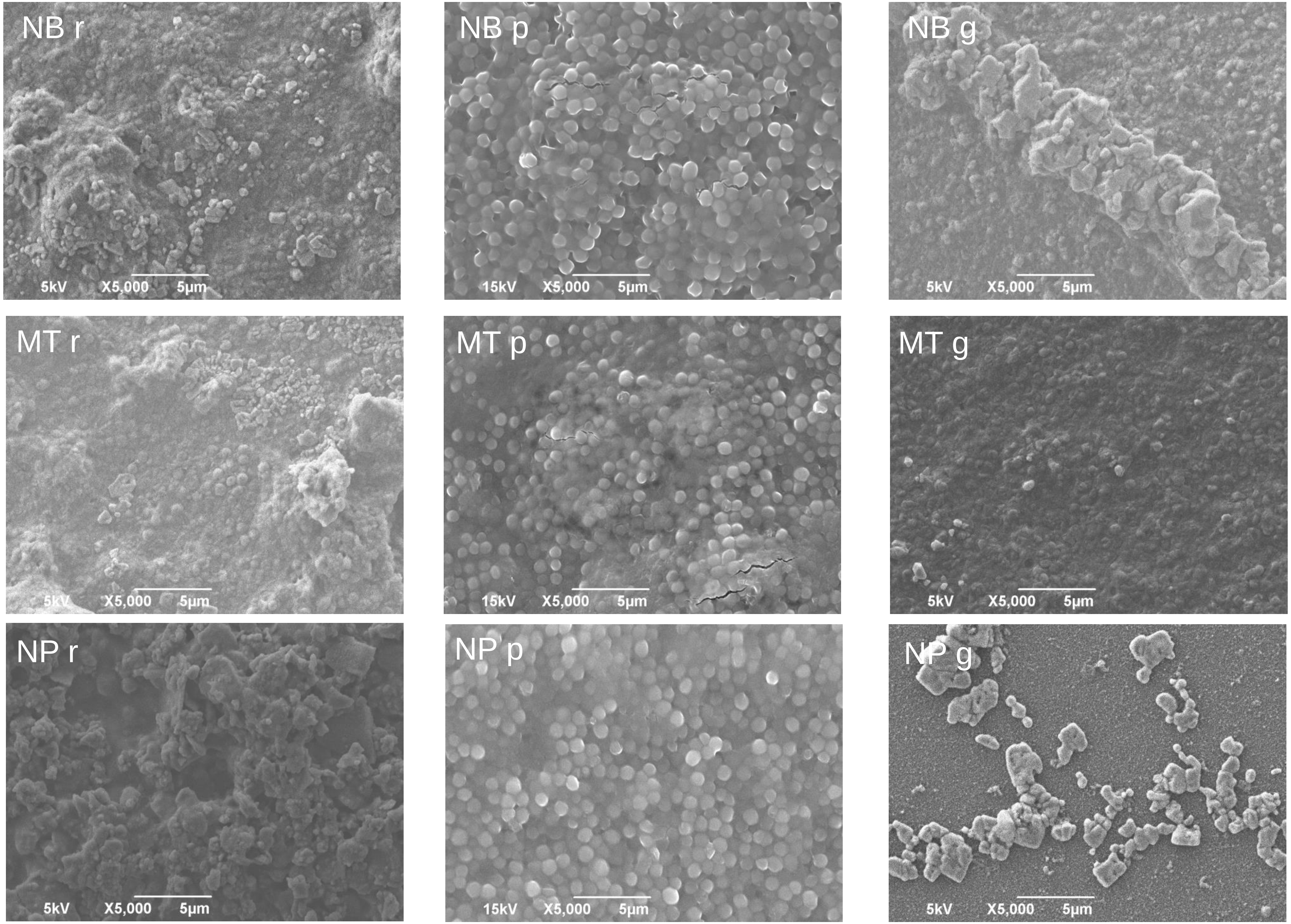

C. albicans
biofilm on disks from:

NB-
Nexdent MFH
bleach

MT-
Mazic DTemp

NP-
Nexdent 3D plus

r - raw;
p - polished;
g - glazed

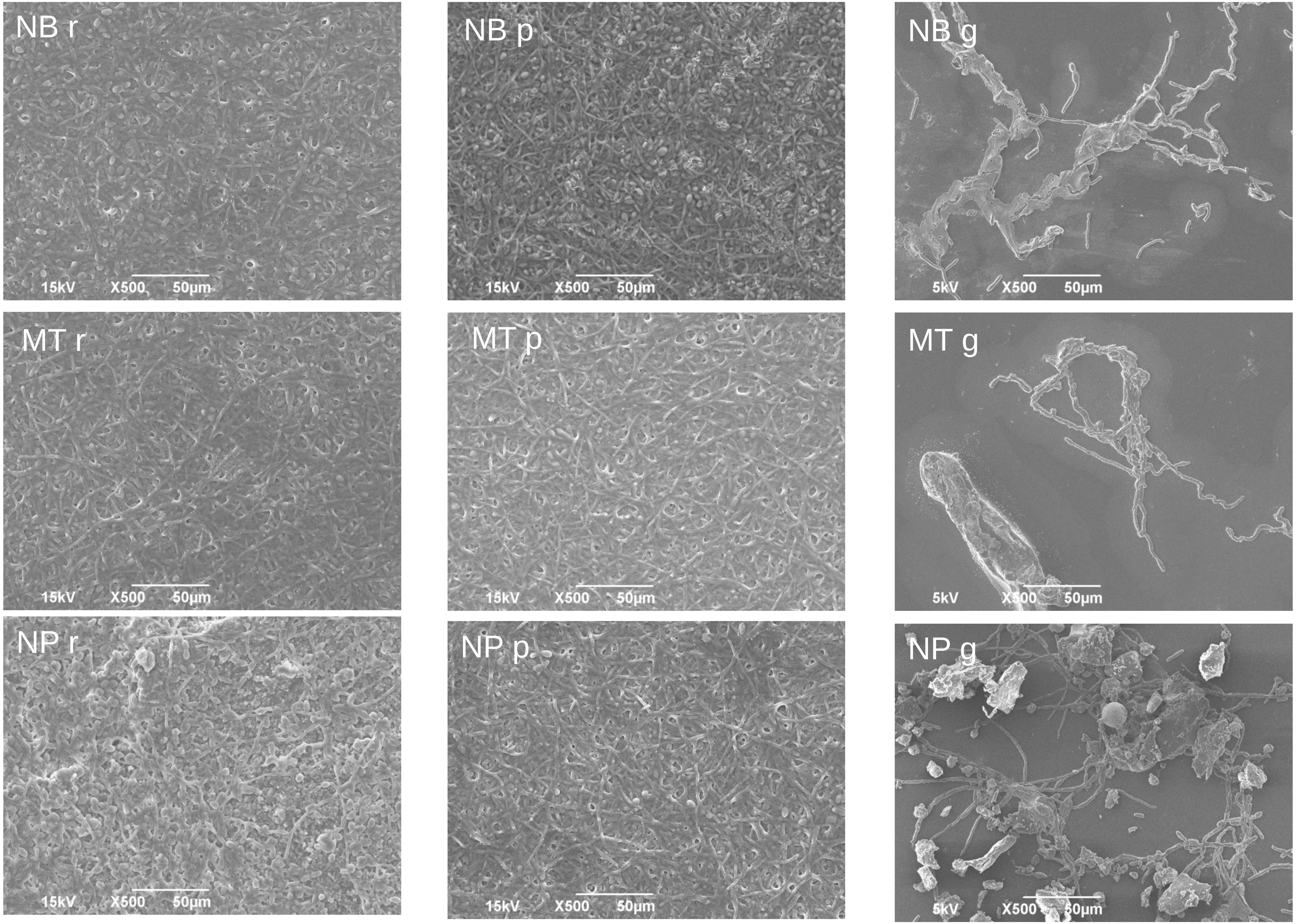

Supplement: Supplementary file 1 — Additional file 1. Supplementary Figure 1. Scanning electron microscopic photographs of microbial biofilms on resins disks. [file 12903_2022_2488_MOESM1_ESM.pdf]
